# Knots Untie: Molecular Determinants Involved in Knot Formation Induced by *Pseudomonas savastanoi* in Woody Hosts

**DOI:** 10.3389/fpls.2017.01089

**Published:** 2017-06-21

**Authors:** Eloy Caballo-Ponce, Jesús Murillo, Marta Martínez-Gil, Alba Moreno-Pérez, Adrián Pintado, Cayo Ramos

**Affiliations:** ^1^Área de Genética, Facultad de Ciencias, Instituto de Hortofruticultura Subtropical y Mediterránea ‘La Mayora’, Universidad de Málaga–Consejo Superior de Investigaciones CientíficasMálaga, Spain; ^2^Departamento de Producción Agraria, ETS de Ingenieros Agrónomos, Universidad Pública de NavarraPamplona, Spain

**Keywords:** bacterial gall, olive knot, woody host, *Pseudomonas savastanoi*, *Pseudomonas syringae*, IAA, cytokinins, T3SS

## Abstract

The study of the molecular basis of tree diseases is lately receiving a renewed attention, especially with the emerging perception that pathogens require specific pathogenicity and virulence factors to successfully colonize woody hosts. Pathosystems involving woody plants are notoriously difficult to study, although the use of model bacterial strains together with genetically homogeneous micropropagated plant material is providing a significant impetus to our understanding of the molecular determinants leading to disease. The gammaproteobacterium *Pseudomonas savastanoi* belongs to the intensively studied *Pseudomonas syringae* complex, and includes three pathogenic lineages causing tumorous overgrowths (knots) in diverse economically relevant trees and shrubs. As it occurs with many other bacteria, pathogenicity of *P. savastanoi* is dependent on a type III secretion system, which is accompanied by a core set of at least 20 effector genes shared among strains isolated from olive, oleander, and ash. The induction of knots of wild-type size requires that the pathogen maintains adequate levels of diverse metabolites, including the phytohormones indole-3-acetic acid and cytokinins, as well as cyclic-di-GMP, some of which can also regulate the expression of other pathogenicity and virulence genes and participate in bacterial competitiveness. In a remarkable example of social networking, quorum sensing molecules allow for the communication among *P. savastanoi* and other members of the knot microbiome, while at the same time are essential for tumor formation. Additionally, a distinguishing feature of bacteria from the *P. syringae* complex isolated from woody organs is the possession of a 15 kb genomic island (WHOP) carrying four operons and three other genes involved in degradation of phenolic compounds. Two of these operons mediate the catabolism of anthranilate and catechol and, together with another operon, are required for the induction of full-size tumors in woody hosts, but not in non-woody micropropagated plants. The use of transposon mutagenesis also uncovered a treasure trove of additional *P. savastanoi* genes affecting virulence and participating in diverse bacterial processes. Although there is still much to be learned on what makes a bacterium a successful pathogen of trees, we are already untying the knots.

## Knot, Knot, Who’s There?

Bacterial galls are characterized by tumorous overgrowths, also known as knots, produced on the stems, leaves, and roots of plants infected with certain bacterial phytopathogens and constitute an important group of plant diseases that cause serious reductions in crop yields and considerable economic losses (Agrios, 2005). Records of bacterial galls go back many centuries; Theophrastus’ description of the formation of knots on the trunks and branches of olive trees in the fourth century B.C. is likely one of the first clear descriptions of plant diseases (Iacobellis, 2001). In contrast, the causal relationship between bacteria and plant galls was not clearly established until the end of the nineteenth century by the Italian plant pathologists Luigi Savastano and Fridiano Cavara; they determined the bacterial etiologyof olive knot disease (*Pseudomonas savastanoi* pv. savastanoi) (Pierce, 1891) and crown gall of grape (*Agrobacterium tumefaciens*) (Cavara, 1897). From then, several bacterial species and genera have been demonstrated to cause galls on plants.

*Agrobacterium tumefaciens* and related species, the only gall-inducing bacterial species that transfer and integrate a fragment of its own DNA (i.e., the T-DNA), into the plant genome (Chilton et al., 1977), cause galls at the base of the trunk and roots on a wide range of woody plant species (Gelvin, 2003). Over 90 families of plants have been found to be susceptible to crown gall disease. However, the bacteria are primarily found in nature on woody plants, such as stone fruits, pome fruits, willows, and grapes (Gelvin, 2003; Pulawska, 2010). Due to their unique mode of action, these species of bacteria have been converted into both a relevant model for the study of plant–bacteria interactions and a biotechnological tool for plant breeding and transformation.

Carrot bacterial gall, first tentatively attributed to *A. tumefaciens* or to the root knot nematode *Meloidogyne* spp., is caused by distinctive Gram-negative bacteria, *Rhizobacter dauci*, which were not recorded in plant bacteriology until the end of the 1980s. Infected plants develop galls along the entire length of the storage roots from the crown to the root tip (Goto and Kuwata, 1988). *R. dauci* appears to have an extremely wide host range and produces galls on the roots, stems and tubers of at least 46 plant species from 24 families, including relevant vegetables such as tomato and cabbage as well as many common weeds (Kawarazaki et al., 2009, 2012).

*Pantoea agglomerans*, a species of commensal bacteria associated with many plant species, has evolved into a host-specific gall-forming pathogen by acquiring a plasmid-borne pathogenicity island. The best-known strains belong to *P. agglomerans* pathovars betae and gypsophilae. While *P. agglomerans* pv. betae elicits gall formation in both gypsophila (*Gypsophila paniculata* L) and beet (*Beta vulgaris* L.), *P. agglomerans* pv. gypsophilae induces symptoms only in gypsophila (Barash and Manulis-Sasson, 2007). Interestingly, *P. agglomerans* strains inhabiting olive trees as harmless endophytes have been shown to cooperate with strains of *P. savastanoi*, forming a stable bacterial consortium that increases the severity of olive knot disease (Hosni et al., 2011).

*Rhodococcus fascians*, an actinomycete that induces the formation of tissue hyperplasia (i.e., leafy gall syndrome), is the only described species of Gram-positive (+) bacteria that causes plant galls. *R. fascians* infects a wide range of plants, primarily dicotyledonous herbs; it initiates the formation of leafy galls consisting of amplified shoot centers and inhibits shoot growth. Because of similarities in disease symptoms and the host range, it was hypothesized that *R. fascians* might be the Gram-positive counterpart of *A. tumefaciens*. However, transfer of bacterial DNA to the plant genome could not be demonstrated for this bacterium (Goethals et al., 2001).

Although diverse gall-forming bacteria exhibit common features during infection and symptom development, such as the production of phytohormones, different bacteria use distinctive systems to produce galls. In this review, we focus on the molecular mechanisms involved in tumor formation by a model bacterial pathogen of woody hosts, *P. savastanoi*.

## Tumorigenic Pathovars of *P. savastanoi*

The species *P. savastanoi* is a member of the *Pseudomonas syringae* complex and includes four pathovars causing knots or excrescences in woody hosts. These are *P. savastanoi* pv. savastanoi (Psv), pv. nerii (Psn), pv. fraxini (Psf) and pv. retacarpa (Psr), comprising isolates from olive (*Olea europaea*), oleander (*Nerium oleander*), ash (*Fraxinus excelsior*) and Spanish broom (*Retama sphaerocarpa*), respectively (Gardan et al., 1992a; Bull et al., 2010) (**Table 1**). Additionally, DNA-DNA hybridization studies formally classified the causative agents of bacterial brown spot of soybeans and halo blight disease of beans into the species *P. savastanoi* (i.e., *P. savastanoi* pv. glycinea and *P. savastanoi* pv. phaseolicola, respectively) (Gardan et al., 1992a). However, it is now clear that *P. savastanoi* belongs to a larger phylogenetic group, which should be called *P. amygdali* when elevated to the species rank, comprising 25 pathovars and four additional *Pseudomonas* species, and that pathovars Psv, Psn, Psf, and Psr are phylogenetically closer to other pathovars infecting trees than to pv. glycinea and pv. phaseolicola (Gardan et al., 1999; Nowell et al., 2014; Baltrus et al., 2017). Remarkably, this phylogenetic group, also known as genomospecies 2 (Gardan et al., 1999) and phylogroup 3 (Baltrus et al., 2017), is the only one from the *P. syringae* complex containing bacteria causing tumors in woody hosts, namely Psv, Psn, Psr, *P. meliae*, *P. tremae* and *P. syringae* pathovars cerasicola, daphniphylli, dendropanacis, myricae, and rhaphiolepidis (Lamichhane et al., 2014).

**Table 1:** Pathovar assignation of *P. savastanoi* strains isolated from diverse woody hosts.

Host family	Host genera/species	Common name	Pathovar^a^	Reference
*Apocynaceae*	*Mandevilla* spp.	Dipladenia	NA	Eltlbany et al., 2012
	*Nerium oleander*	Oleander	nerii	Janse, 1982
*Fabaceae*	*Retama sphaerocarpa*	Spanish broom	retacarpa	Alvarez et al., 1998
*Lythraceae*	*Punica granatum*	Pomegranate	savastanoi^b^	Bozkurt et al., 2014
*Myrtaceae*	*Myrtus communis*	Myrtle	NA	Temsah et al., 2007b
*Oleaceae*	*Fontanesia phillyreoides*	Fontanesia	savastanoi^b^	Mirik et al., 2011
	*Forsythia* spp.	Forsythia	NA	Bradbury, 1986
	*Fraxinus excelsior*	Ash	fraxini	Janse, 1982
	*Jasminum* spp.	Jasmine	NA	Gardan et al., 1992b
	*Ligustrum* spp.	Privet	NA	Gardan et al., 1992b
	*Olea europaea*	Olive	savastanoi	Janse, 1982
	*Osmanthus fragrans*	Sweet olive	NA	Cinelli et al., 2013
	*Phillyrea* spp.	Mock privet	NA	Gardan et al., 1992b
*Rhamnaceae*	*Rhamnus alaternus*	Buckthorn	NA	Temsah et al., 2007a


*^a^NA, not assigned.*

*^b^For fontanesia and pomegranate isolates, the assignation to pv. *savastanoi* was based in biochemical tests, fatty acid profiling, and PCR.*

While artificial infections with Psv, Psn, and Psr strains cause knots, Psf isolates induce cankers accompanied by wart-like excrescences in both ash and olive. Psv strains induce knot formation in olive and ash but not in oleander, whereas Psn strains can infect all three hosts. In contrast, the host range of Psr isolates is restricted to Spanish broom (Janse, 1982; Iacobellis et al., 1998; Ramos et al., 2012). Knot formation by *P. savastanoi* has also been described in several other hosts, including jasmine (Gardan et al., 1992b), privet (Gardan et al., 1992b), mock privet (Gardan et al., 1992b), forsythia (Bradbury, 1986), buckthorn (Temsah et al., 2007a), fontanesia (Mirik et al., 2011), pomegranate (Bozkurt et al., 2014), myrtle (Goumas et al., 2000; Temsah et al., 2007b; Cinelli et al., 2014) and dipladenia (Putnam et al., 2010; Eltlbany et al., 2012; Pirc et al., 2014; Caballo-Ponce and Ramos, 2016) (**Table 1**). Although these *P. savastanoi* hosts are classified in several plant families, over 50% of them belong to the *Oleaceae* family. Lack of accurate cross-pathogenicity tests for most of these strains preclude their classification within any of the four well-established *P. savastanoi* pathovars causing diseases in woody hosts (**Table 1**).

Knot induction by *P. savastanoi* in woody hosts is characterized by the formation of hypertrophic and hyperplastic overgrowths on the trunks, stems and branches; however, this symptomatology is rarely observed on the leaves and fruits (Janse, 1980; Rodríguez-Moreno et al., 2008, 2009; Mirik et al., 2011; Eltlbany et al., 2012; Ramos et al., 2012; Bozkurt et al., 2014). Considerable progress in identifying and understanding bacterial mechanisms that govern knot generation by *P. savastanoi*, some of which are specific to woody host pathogens, has been achieved in recent years (**Table 2**) and will be reviewed below.

**Table 2:** Described mutations affecting virulence and pathogenicity factors in tumorigenic pathovars of *Pseudomonas savastanoi.*

Trait affected	Gene	Pathovar	Function	Effect in knot development	Other phenotypes	Reference
Metabolism of IAA	p-IAA^a^	savastanoi/nerii	IAA biosynthesis	Absence of knot	Enhanced swarming	Comai and Kosuge, 1980; Surico et al., 1985; Soby et al., 1991
	*iaaMH*^b^	savastanoi	IAA biosynthesis	Absence of knot		Aragón et al., 2014
	*iaaM*	nerii	IAA biosynthesis	Absence of knot		Cerboneschi et al., 2016
	*iaaL*	nerii	IAA conjugation	Volume reduction		Glass and Kosuge, 1988
	*iaaL*	nerii	IAA conjugation	Volume increase		Cerboneschi et al., 2016
Production of cytokinins	Δp-*ptz*^c^	nerii	Cytokinin biosynthesis	Volume reduction		Iacobellis et al., 1994
	ΔpPsv48A^c^	savastanoi	Cytokinin biosynthesis	Volume reduction		Bardaji et al., 2011
Type III secretion system	*hrcC*	savastanoi	Outer membrane pore protein	Absence of knot	No HR^d^ in *Nicotiana tabacum*	Sisto et al., 2004
	*hrpA*	savastanoi	Pilin protein	Absence of knot	Unable to multiply in plant tissue No HR^d^ in *Nicotiana tabacum*	Pérez-Martínez et al., 2010
		nerii	Pilin protein	Absence of knot		Cerboneschi et al., 2016
	*hrpR*	savastanoi	Transcriptional regulator	Absence of knot	Unable to multiply in plant tissue	Matas et al., 2012
	*hrpL*	savastanoi	Transcriptional regulator (sigma-54)	Absence of knot	No HR^d^ in *Nicotiana tabacum*	Matas et al., 2014
	*hopAO1*	savastanoi	Phosphatase	Volume reduction Increased necrosis	Reduced competitiveness in olive plants	Castañeda-Ojeda et al., 2017b
c-di-GMP metabolism	*bifA*	savastanoi	Phosphodiesterase	Volume reduction	Reduced swimming	Aragón et al., 2015a
	*dgcP*	savastanoi	Diguanylate cyclase	Volume reduction	Increased swimming Reduced biofilm formation Decreased expression of T6SS genes (*hcp1* and *vgrG*)	Aragón et al., 2015b
	*pleD*^e^	savastanoi	Diguanylate cyclase	Volume increase Reduction of necrotic tissue	Reduced swimming Increased biofilm formation	Perez-Mendoza et al., 2014
Quorum sensing	*luxI*	savastanoi	AHL synthase	Volume reduction	Reduced exopolysaccharide production	Hosni et al., 2011
	*luxR*	savastanoi	AHL receptor	Volume reduction	Reduced exopolysaccharide production	Hosni et al., 2011
Metabolism of phenolics	*antA*	savastanoi	Anthranilate catabolism	Volume reduction		Caballo-Ponce et al., 2017
	*catB*	savastanoi	Catechol catabolism	Volume reduction		Caballo-Ponce et al., 2017
	*ipoABC*^b^	savastanoi	Oxygenase on phenolics	Volume reduction		Caballo-Ponce et al., 2017


*^a^Analyzed in strains cured of a plasmid encoding the *iaaMH* operon.*

*^b^Genes *iaaM-iaaH* and *ipoA-ipoB-ipoC* are organized in operons.*

*^c^Analyzed in strains cured of a plasmid encoding the *ptz* gene.*

*^d^HR, hypersensitive response.*

*^e^Overexpression of *pleD* from *Caulobacter crescentus.**

## *P. savastanoi*-Induced Knots: A Modified Tissue Structure Connected With the stem Vascular System

**FIGURE 1:**
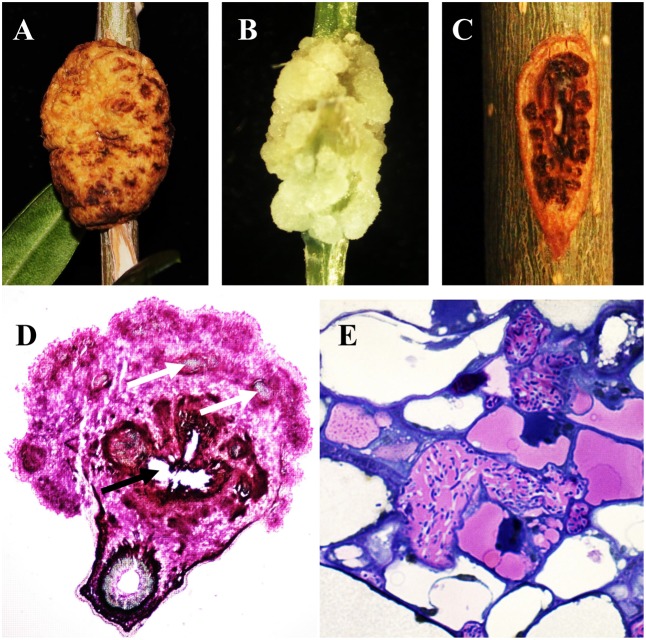
Symptoms induced by *Pseudomonas savastanoi* and pathogen visualization within knots. Knots induced by Psv NCPPB 3335 in **(A)** woody olive trees at 90 days post-inoculation (dpi) and **(B)** micropropagated (non-woody) olive plants at 28 dpi. **(C)** Excrescence-like symptoms generated by Psf NCPPB 1006 at 90 dpi in ash. **(D)** Transverse section of a 30-dpi olive knot stained with xylene blue-pyrofuchsin. White and black arrows indicate newly formed xylem vessels and cavities within the knot, respectively. **(E)** Detail of bacterial cavity within a 35-dpi olive knot filled with Psv cells.

The life cycle of *P. savastanoi* has been most extensively characterized for olive isolates. Psv lives epiphytically on the surface of leaves and stems and reaches the highest cell populations in warm, rainy months (Ercolani, 1978; Quesada et al., 2007). However, these bacteria are able to switch from an epiphytic to an endopathogenic lifestyle after penetration into plant tissues through wounds generated by climatic conditions or agricultural practices. As a pathogen, *P. savastanoi* induces hypertrophy and hyperplasia of plant tissues, generally leading to the generation of knots (**Figures 1A,B**), an exception occurs in ash infections by Psf strains, which induce the formation of small protuberances (i.e., cankers or excrescences) (**Figure 1C**). A histological examination of the knots induced by *P. savastanoi* infections in different hosts reveals strong similarities among olive (Surico, 1977; Temsah et al., 2008; Rodríguez-Moreno et al., 2009), oleander (Wilson, 1965; Temsah et al., 2010), buckthorn (Temsah et al., 2007a), and myrtle (Temsah et al., 2007b). During the early stages of an infection, bacteria are localized in the intercellular spaces of the cortical parenchyma and in the vascular tissues damaged by the wound, where bacterial pectolytic and hemicellulolytic enzymatic activities cause cell wall degradation of adjacent plant cells, resulting in the plasmolysis of host cells and the generation of internal cavities. Bacterial cells colonize these cavities and multiply, while lignin deposits appear as a defense mechanism in the cell walls of plasmolyzed cells. Adjacent to them, the parenchymal cells of the cortex and vascular system show hypertrophic and hyperplastic activities initiated by coenocytic divisions, resulting in multinucleated cells. Additionally, in oleander infections, tyloses develop in the xylem vessels, hampering bacterial movement (Temsah et al., 2010).

Subsequent enlargement of the internal cavities and hyperplastic tissues result in the formation of an incipient multilobed knot. Additionally, the parenchyma cells adjacent to the bacterial cavities have been shown to differentiate into xylem elements (**Figure 1D**), which are connected with the primary vascular cylinder in olive infections (Rodríguez-Moreno et al., 2009). Taking into account that neovascularization is essential for the growth of plant and animal tumors (Ullrich and Aloni, 2000), the existence of this network of vascular tissues might facilitate the supply of water and nutrients necessary for the knots to reach their final size. The existence of a vascular connection between plant galls and the vascular system of the host plant has also been reported in *A. tumefaciens*-induced crown galls (Aloni et al., 1995).

During later stages of knot development, the hypertrophied tissue is composed of groups of disorganized cells, except for those being part of the xylem vessels generated during the infection. Psv cells have been visualized inside olive knots forming small groups and microcolonies, which remain attached to the surface of hypertrophied host cells and to each other by an extracellular matrix. However, most bacterial cells are found forming multilayer biofilms localized within the internal cavities of the neoplastic tissue (**Figure 1E**), and surrounding the stem vascular system. In olive knots, Psv cells have also been visualized in the lumen of newly formed xylem vessels, suggesting that the pathogen might migrate through the vascular system (Rodríguez-Moreno et al., 2009; Maldonado-González et al., 2013). In oleander infections, Psn cells have been visualized in xylem vessels and also in laticifers, where they multiply and spread systemically (Wilson and Magie, 1964). In addition, the pressure generated by continuous hyperplastic activity causes the appearance of fissures in the knot tissues. The enlargement of these fissures produces breakage of the periderm and exposure of the internal cavities filled with bacteria to the environment, which contributes to pathogen spread.

## Pathogen-Produced Phytohormones and their Role in Disease

Phytohormones have a central role in plant defenses against abiotic and biotic stresses such as drought, herbivores and pathogens (O’Brien and Benková, 2013; Ma and Ma, 2016). It is thus not surprising that many types of pathogens evolved diverse strategies to interfere with the biosynthesis or activity of phytohormones, both to dampen plant immunity and to obtain shelter and nutrients (Boivin et al., 2016; Ma and Ma, 2016). However, it is their balance that ultimately modulates plant responses; thus, many non-pathogenic bacteria also synthesize phytohormones, which contributes to enhanced plant growth and health (Arkhipova et al., 2007; Kudoyarova et al., 2014).

### Indole-3-Acetic Acid, a *P. savastanoi* Pathogenicity Determinant for Knot Formation

The auxin phytohormone indole-3-acetic acid (IAA) is required for growth and development of plant tissues (Santner and Estelle, 2009; Kieffer et al., 2010). However, bacterial production of IAA can interfere with plant development by disturbing the auxin balance in plants (Spaepen and Vanderleyden, 2011). Furthermore, biosynthesis of IAA has been described as a pathogenicity or virulence factor in bacterial phytopathogens (Patten et al., 2013). Redundancy in IAA biosynthesis is widespread among plant-associated bacteria, and several IAA biosynthetic pathways have been described, most of which are dependent on L-tryptophan as a precursor (Spaepen et al., 2007). Production of IAA by phytopathogenic bacteria via the indole-3-acetamide pathway was first characterized in the gall-forming pathogens *A. tumefaciens* and *P. savastanoi* (Smidt and Kosuge, 1978; Thomashow et al., 1984). In *P. savastanoi*, tryptophan is initially converted into indole-3-acetamide by tryptophan 2-monooxygenase (encoded by the *iaaM* gene) and later transformed into the final product, IAA, in a reaction catalyzed by indole-3-acetamide hydrolase (encoded by the *iaaH* gene) (Magie et al., 1963; Kosuge et al., 1966). Organization of the *iaaM* and *iaaH* genes as an operon (*iaaMH*) has been described in several *P. savastanoi* strains (Comai and Kosuge, 1980; Palm et al., 1989; Comai et al., 1992). However, while most Psv strains encode the *iaaMH* operon on the chromosome, Psn isolates usually harbor these genes on plasmids (Glass and Kosuge, 1986; Caponero et al., 1995; Pérez-Martínez et al., 2008). In addition, subsequent analyses have revealed that Psv strains frequently encode two copies of the *iaaMH* operon (named *iaaMH1* and *iaaMH2*), both of which are located on the chromosome (Pérez-Martínez et al., 2008; Rodríguez-Palenzuela et al., 2010). In the model Psv strain NCPPB 3335, the *iaaM2* gene encodes an internal insertion of 22 nucleotides. In addition, a knock-out *iaaMH2* mutant of this strain produces similar amounts of IAA as the wild-type strain, suggesting that this extra copy of the operon is not functional (Aragón et al., 2014). Interestingly, single- (*iaaMH1*) and double- (*iaaMH1* and *iaaMH2*) deletion mutants of Psv NCPPB 3335 produce residual amounts of IAA, suggesting that redundancy in the IAA biosynthetic pathways also occurs in *P. savastanoi* (Aragón et al., 2014).

**FIGURE 2:**
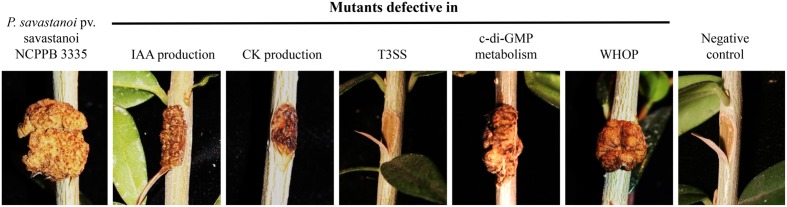
Knots induced by Psv NCPPB 3335 and derivative strains with altered virulence or pathogenicity in woody olive plants at 90 dpi. Pictures correspond to typical symptoms induced by the wild-type strain (NCPPB 3335) and derivative mutants for genes *iaaMH1* (IAA production), *hrpR* (T3SS), *dgcP* (c-di-GMP metabolism) and *antA* (WHOP), as well as a Psv NCPPB 3335 derivative cured of pPsv48A, a plasmid encoding the biosynthetic gene *ptz*, and defective in cytokinins (CKs) production. Negative control, plant inoculated with sterile MgCl_2_.

The role of IAA production in the pathogenicity of Psv and Psn isolates has been examined using different approaches. First, Psv and Psn strains resistant to the growth inhibitor α-methyltryptophan and showing an altered production of IAA were isolated. While Psv and Psn IAA-deficient mutants failed to cause knot development in olive and oleander plants, respectively, Psn strains overproducing this phytohormone formed larger knots than the wild-type strain (Smidt and Kosuge, 1978; Surico et al., 1985), thus suggesting the role of this phytohormone in the pathogenicity of *P. savastanoi*. A different tryptophan analog, 5-methyltryptophan, has been used for the isolation of *P. savastanoi* strains cured of *iaaM*-encoding plasmids. This strategy is based on the toxicity of 5-methyltryptophan for *P. savastanoi* mutants lacking tryptophan 2-monooxygenase activity (i.e., *iaaM* gene). Thus, *P. savastanoi* mutants sensitive to 5-methyltryptophan and lacking the *iaaM* gene were selected upon treatment of bacterial cells with the DNA-intercalating agent acridine orange (Comai and Kosuge, 1980). Although Psn cells without *iaaM*-encoding plasmids were able to induce necrosis in the tissues into which they were inoculated, they failed to generate knots in oleander plants (Iacobellis et al., 1994). Construction of the single *iaaMH1* and the double *iaaMH1 iaaMH2* mutants mentioned above in Psv NCPPB 3335 provided direct evidences for the involvement of this operon in Psv pathogenicity. As expected from their low IAA levels, knot induction by these two mutant strains was fully abolished, further supporting the role of IAA as a pathogenicity determinant in this bacterial pathogen (Aragón et al., 2014) (**Figure 2**).

In addition to IAA production, *P. savastanoi* might modulate the pool of free IAA by transforming it into a less biologically active amino acid conjugate, 3-indole-acetyl-ε-L-lysine (IAA-Lys), through activity encoded in the *iaaL* gene (Hutzinger and Kosuge, 1968; Glass and Kosuge, 1986). The number of copies and the location of the *iaaL* gene differ among *P. savastanoi* pathovars. While Psv strains generally harbor two *iaaL* paralogs, both located in the chromosome, Psn strains usually encode the *iaaL* gene in plasmids (Matas et al., 2009). Furthermore, IAA-Lys has been detected in culture filtrates of Psn isolates, whereas this compound could not be detected in culture supernatants of Psv strains (Evidente et al., 1985). Bacteria from the *P. syringae* complex, including *P. savastanoi*, encode a putative multidrug and toxic compound extrusion transporter of the MATE family (*matE* gene) upstream of the *iaaL* gene. These genes have been shown to be transcribed either independently or as part of an operon in *P. syringae* pv. tomato DC3000. Interestingly, deletion of either of these two genes resulted in reduced fitness and virulence of DC3000 in tomato plants (Castillo-Lizardo et al., 2015). Nonetheless, the role of *iaaL* in the virulence of *P. savastanoi* seems to be dependent on the strain and the experimental conditions used. A Psn PB213 *iaaL* mutant obtained by Tn*5* transposition displayed virulence attenuation in woody oleander plants in comparison with the wild-type strain (Glass and Kosuge, 1988). Conversely, a recent report showed that a knockout mutation of the *iaaL* gene in a different Psn isolate, Psn23, yielded a hypervirulent strain that resulted in a severe increase in knot size, higher systemic dispersion and a larger final population size than the wild-type strain in micropropagated (i.e., non-woody) oleander plants (Cerboneschi et al., 2016). Further studies are necessary to clarify the role of the *iaaL* gene in the virulence of *P. savastanoi* strains isolated from different hosts.

### Production of Cytokinins by *P. savastanoi* Contributes to the Development of Mature Xylem Vessels within the Knots

Cytokinins (CKs) are derivatives of adenine nucleotides with an isoprene-derived or aromatic side chain in position *N^6^* (Sakakibara, 2006); they have many diverse functions including control of various processes in plant growth and development and modulation of plant defenses against stresses (Sakakibara, 2006; O’Brien and Benková, 2013). *P. savastanoi* strains from olive and oleander produce at least zeatin, dihydrozeatin, 1’-methyl-zeatin, ribosylzeatin, ribosyldihydrozeatin, and ribosyl-1”-methylzeatin, as well as diverse other methylated zeatin derivatives; however, the types and amounts of CKs types vary among strains (Surico et al., 1985; Evidente et al., 1986; MacDonald et al., 1986; Iacobellis et al., 1994).

The *ptz* gene (*Pseudomonas* trans-zeatin producing gene, also called *ipt*; 705 nt), coding for an isopentenyl transferase (234 aa), is widely present in tumorigenic strains of *P. savastanoi* (Ramos et al., 2012). Thus, isolates of Psf inducing cankers, but not knots, on ash do not possess the *ptz* gene (Iacobellis et al., 1998). The deduced product of *ptz* catalyzes the prenylation of adenosine 5’-phosphates (AMP, ADP, or ATP) with either dimethylallyl diphosphate or hydroxymethylbutenyl diphosphate, a main step in the isoprenoid CKs biosynthesis (Sakakibara, 2006). Although preceded by and in tight association with another gene, *ptz* appears to have its own promoter and, when expressed in *E. coli*, directs the biosynthesis of CKs (MacDonald et al., 1986; Powell and Morris, 1986), suggesting that it is the only specific gene from *P. savastanoi* responsible for CKs biosynthesis. The gene shows only approximately 50% nucleotide identity with the homologous *tmr* and *tzs* genes from *A. tumefaciens*, with similar levels of identity among the corresponding products. The *ptz* gene is found in plasmid pPsv48A (PSPSV_A0024) of strain Psv NCPPB 3335 and in the chromosome or plasmids in other strains of Psv and Psn (Caponero et al., 1995; Pérez-Martínez et al., 2008; Ramos et al., 2012). Likewise, it is present in the draft genome of the tumorigenic Psr strain ICMP16945 (accession no. LJRD01000455).

The *idi* gene (previously called *ipt*; isopentenyl-diphosphate delta isomerase, 152 aa) is found in plasmid pPsv48C from Psv NCPPB 3335 and might also be involved in CKs biosynthesis. Its deduced product catalyzes the conversion of isopentenyl pyrophosphate to dimethylallyl diphosphate, which is a key step in biosynthesis of CKs through the mevalonate pathway (Sakakibara, 2006). However, homologs of this gene are present in a wide variety of bacteria, including *E. coli*, and it is possible that it carries functions other than or in addition to the biosynthesis of CKs.

The involvement of CKs in the pathogenicity and virulence of tumorigenic pseudomonads is supported by several lines of evidence. Early work already showed that the amount of IAA and CKs produced in culture by Psv and Psn strains was positively correlated with the production of larger knots even after the shortest incubation periods (Surico et al., 1985). Likewise, knots in olive plants caused by *P. savastanoi* strains without plasmids containing the *ptz* gene were smaller (**Figure 2**) and had fewer spiral vessels than knots induced by wild-type strains (Iacobellis et al., 1994; Rodríguez-Moreno et al., 2008; Bardaji et al., 2011). However, mutants that were unable to synthesize CKs reached similar populations sizes as the corresponding wild-type strains in oleander leaves (Iacobellis et al., 1994) and in olive tissues (Rodríguez-Moreno et al., 2008; Bardaji et al., 2011). Thus, CKs appear to be non-essential for tumor formation but to contribute to full expression of disease symptoms. Nevertheless, because previous assays were done with mutants obtained by plasmid curing, to precisely assess the contribution of CKs to the bacterial life cycle, it will be necessary to evaluate the behavior of gene-specific mutants by themselves and in competition with wild-type strains.

### Phytohormone Crosstalk with Other Virulence Factors

Regulatory networks involving crosstalk between phytohormones and other pathogenicity and virulence factors have been described in several plant pathogens, including the gall-forming bacterium *P. agglomerans* pv. gypsophilae (Chalupowicz et al., 2009). Biosynthesis of IAA in this pathogen takes place by two different pathways, the indole-3-acetamide and the indole-3-pyruvate routes, which differentially contribute to gall formation and epiphytic fitness, respectively (Manulis et al., 1998). While inactivation of the biosynthesis of either CKs or IAA through the indole-3-acetamide pathway led to repression of the type III secretion system (T3SS) and the quorum sensing system, transcriptional upregulation of these two systems was observed in a mutant unable to synthesize IAA via the indole-3-pyruvate pathway (Chalupowicz et al., 2009). Similarly, transcriptional downregulation of the T3SS, the global regulatory gene *gacA* and the pectate lyase genes *pelD*, *pelI*, and *pelL* has been reported in a *Dickeya dadantii* (formerly known as *Erwinia chrysanthemi*) *iaaM* mutant (Yang et al., 2007).

Beyond the direct role of IAA in knot induction by *P. savastanoi*, only a few studies have suggested that this molecule might also function as a signaling molecule in this bacterium. Psn IAA-deficient mutants exhibit enhanced swarming motility (**Table 2**) (Soby et al., 1991), indicating that the internal IAA pool influences *P. savastanoi* motility. However, the expression of virulence-related genes in Psv mutants affected in the biosynthesis of CKs or IAA has not yet been analyzed. On the other hand, exogenous addition of IAA to a Psv NCPPB 3335 culture caused repression of the T3SS genes *hrpL* and *hrpA*, whereas transcription of the type VI secretion system (T6SS) gene *vgrG* was activated (Aragón et al., 2014). These results, which are in accord with the repression of the *vir* regulon reported in *A. tumefaciens* cells exposed to exogenous IAA (Yuan et al., 2008), suggest an additional effect of IAA during the interaction of *P. savastanoi* with its hosts that deserves further exploration. Additionally, a *ptz* homolog gene and/or CKs appear to regulate the expression of diverse virulence genes in *A. tumefaciens* (Hwang et al., 2013). For this reason and because there is evidence that a plethora of beneficial and pathogenic microorganisms modulate the phytohormone pool (Boivin et al., 2016), it is possible that IAA and CKs contribute to the life cycle of *P. savastanoi* in other, more subtle, unexplored ways.

## Knot Formation By *P. savastanoi* Requires A Functional Type III Secretion System

The T3SS of pathogenic bacteria infecting animals and plants is responsible for the injection of specialized proteins, called T3SS effectors (T3Es), into the host cytoplasm (Hueck, 1998; Schulmeyer and Yahr, 2017). In bacterial phytopathogens, the T3SS is required for both disease development in susceptible hosts and the triggering of programmed cell death, also called a hypersensitive response (HR), in resistant hosts (Galan and Collmer, 1999; Büttner and He, 2009). Additionally, the T3SS participates in symbiotic *Rhizobium*-legume interactions, in which it is required for effective nodule formation (Fauvart and Michiels, 2008; Büttner, 2012).

In the *P. syringae* complex, the T3SS is organized in a cluster within a pathogenicity island, which is predominantly located in the bacterial chromosome. This system is composed of 27 structural and regulatory genes, referred to as the *hrp* cluster (hypersensitive response and pathogenicity), and organized in five operons (*hrpRS*, *hrpZ*, *hrpC*, *hrpU*, and *hrpJ*) and two independent genes (*hrpK* and *hrpL*) (Alfano et al., 2000; Collmer et al., 2000). Among the *hrp* cluster, a group of genes highly conserved across plants’ and animals’ bacterial pathogens has been found; collectively they are called the *hrc* genes (hypersensitive response and conserved) (Bogdanove et al., 1996). HrpL is one of the most important transcriptional regulators of the T3SS, acting as an activator of the *hrp/hrc* cluster, most of the T3E genes and their corresponding chaperones, among other genes (Xiao and Hutcheson, 1994; Xiao et al., 1994; Mucyn et al., 2014; Waite et al., 2017). In addition to the canonical T3SS, a group of genes forming an incomplete T3SS, similar to that described in *Rhizobium* species, has been found in the genomes of several *P. syringae* and *P. savastanoi* isolates (Rodríguez-Palenzuela et al., 2010; Tegli et al., 2011; Baltrus et al., 2017). However, this secondary T3SS has not been related to pathogenicity, and its function remains unclear (Martínez-García et al., 2015).

Although most studies on the role and structure of the T3SS in strains within the *P. syringae* complex have focused on bacteria isolated from herbaceous hosts, *P. savastanoi* also encodes a complete and functional T3SS, which is phylogenetically related to that of other strains of the *P. syringae* complex (Rodríguez-Palenzuela et al., 2010; Tegli et al., 2011; Matas et al., 2014; Baltrus et al., 2017). Knot formation in olive trees by Psv has been demonstrated to be dependent on the functionality of the T3SS (**Figure 2** and **Table 2**). A Psv ITM 317-derivative mutant of the *hrcC* gene, encoding a structural element of the T3SS, was not able to induce a visible HR in *Nicotiana tabacum* leaves and was impaired in tumor development in olive plants (Sisto et al., 1999, 2004). Similar results have been obtained in woody olive plants for Psv NCPPB 3335 mutants of the *hrpA* gene (Pérez-Martínez et al., 2010), encoding the major structural protein of the Hrp pilus and the transcriptional regulatory genes *hrpR* (Pérez-Martínez et al., 2010; Matas et al., 2012) and *hrpL* (Matas et al., 2014).

**FIGURE 3:**
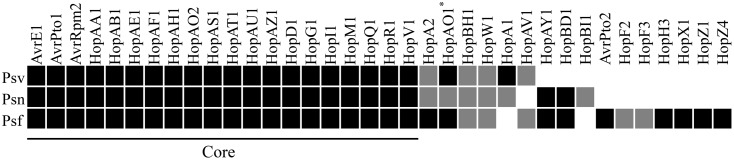
Putative type III effectors (T3Es) identified in Psv NCPPB 3335 (Psv), Psn ICMP 16943 (Psn) and Psf ICMP 7711 (Psf) (Matas et al., 2014; Nowell et al., 2016). Black, gray and white boxes indicate presence, possible truncation and absence, respectively, of T3Es in the genomes of these strains. Core refers to the T3Es common among the three strains (excluding truncated proteins). The presence of five Psv NCPPB 3335 T3Es (HopAF1-2^∗^, HopAH2, HopBK1, HopBL1, and HopBL2) has not been investigated in Psn ICMP 16943 and Psf ICMP 7711. Asterisks correspond to plasmid-encoded effectors in Psv NCPPB 3335.

Even though the mechanisms by which a great number of T3Es interfere with plant immunity remain unknown, the T3E repertoire of phytopathogenic bacteria is one of the most relevant factors in determining host range (Baltrus et al., 2011). *In silico* analyses of several *P. savastanoi* strains have identified a pool of putative T3Es encoded by Psv NCPPB 3335, Psn ICMP 16943 and Psf ICMP 7711, which are composed of 31, 28, and 34 proteins, respectively (**Figure 3**) (Rodríguez-Palenzuela et al., 2010; Ramos et al., 2012; Matas et al., 2014; Nowell et al., 2016). A more thorough characterization of the T3E repertoire has been made in Psv NCPPB 3335, resulting in the demonstration of HrpL-dependent transcription and the translocation to the plant cells of eight proteins that exhibit homology with previously described *P. syringae* T3Es (i.e., AvrRpm2, HopA1, HopAA1, HopAF1-1, HopAF1-2, HopAO1, HopAO2, HopAZ1) and three effectors belonging to two novel T3E families (i.e., HopBK1, HopBL1, and HopBL2) (Matas et al., 2014; Castañeda-Ojeda et al., 2017a,b). Interestingly, *hopBL1* and *hopBL2* sequences have been uniquely identified in a collection of 31 Psv strains isolated in different countries and in other *P. syringae* strains isolated from woody hosts; this indicates a relevant role of the HopBL family in bacterial interactions with olive plants and other trees (Matas et al., 2014). Interference with early plant defense responses has been reported for all these eleven Psv NCPPB 3335 T3E. Furthermore, five of these proteins, HopAZ1, HopAF1-2, HopAO1, HopAO2, and HopBL1, also inhibit effector-triggered immunity (Matas et al., 2014; Castañeda-Ojeda et al., 2017a,b). Moreover, and in agreement with their role in the suppression of early plant defenses, HopAF1-1 and HopAF1-2 have been shown to localize in the plant plasma membrane (Castañeda-Ojeda et al., 2017a). On the other hand, HopAO1 and HopAO2 have been reported to possess phosphatase activity, a distinguishing characteristic of the members of the HopAO family. Deletion of the *hopAO1* gene in NCPPB 3335 resulted in reduced virulence in olive plants (**Table 2**) (Castañeda-Ojeda et al., 2017b). Additional studies are required to identify the complete T3E repertoires of *P. savastanoi* strains isolated from different hosts and to determine their role in regulating knot size and host range.

## Cyclic di-GMP Levels Impact the Motile to Sessile Switch in *P. savastanoi* and Modulate Virulence and Knot Size

Cyclic di-GMP (c-di-GMP), an ubiquitous and well-characterized bacterial secondary messenger, is involved in a wide range of bacterial behaviors, including motility, synthesis and secretion of surface proteins and exopolysaccharides, adhesion to host cells, aggregation, biofilm formation and intracellular infection; these behaviors globally influence bacterial virulence of human, animal, and plant pathogens (Dow et al., 2006; Jenal and Malone, 2006; Tamayo et al., 2007; Lee et al., 2010; Yi et al., 2010; Yang et al., 2012; Huang et al., 2013; Perez-Mendoza et al., 2014). Synthesis of c-di-GMP is mediated by diguanylate cyclases (DGCs), mainly associated with GGDEF domains (Pei and Grishin, 2001), while phosphodiesterases (PDEs) are responsible for the hydrolysis of the secondary molecule through EAL or HD-GYP domains (Ryan et al., 2009; Sultan et al., 2011). C-di-GMP signaling, including characteristic signal-input domains of these enzymes and protein effectors, has been widely discussed previously and have highlighted the significance of environmental signals in c-di-GMP-related phenotypes (Romling et al., 2005; Krasteva et al., 2012; Sondermann et al., 2012; Valentini and Filloux, 2016).

The impact of c-di-GMP in the virulence of plant pathogenic bacteria, including *Xanthomonas campestris*, *Dickeya dadantii*, *Erwinia amylovora*, *Xylella fastidiosa*, *Pectobacterium atrosepticum*, and bacteria from the *P. syringae* complex, has been recently reviewed (Ham, 2013; Ryan, 2013; Martínez-Gil and Ramos, 2017). In *P. syringae* pv. tomato DC3000, high levels of the secondary messenger caused by the DGC Chp8 have been related to active evasion of the plant’s immunity, decreasing flagellin and increasing production of extracellular polysaccharides (Engl et al., 2014; Pfeilmeier et al., 2016). BifA, a conserved PDE previously identified in *P. aeruginosa* (Kuchma et al., 2007; Jimenez-Fernandez et al., 2015), has been connected to flagellar motility, fitness and virulence of this tomato pathogen (Aragón et al., 2015a). Furthermore, a recent study identified AmrZ, a novel transcriptional regulator involved in DC3000 pathogenesis, which controls two putative c-di-GMP metabolic enzymes, AdcA and MorA (Prada-Ramirez et al., 2016).

An *in silico* analysis of the draft genome of Psv NCPPB 3335 revealed the presence of 34 GGDEF- or GGDEF/EAL-containing proteins, which could be related to c-di-GMP metabolism in this phytopathogen (Rodríguez-Palenzuela et al., 2010). The first studies carried out to evaluate the role of c-di-GMP in *P. savastanoi* used the active DGC PleD from *Caulobacter crescentus* (Aldridge et al., 2003; Perez-Mendoza et al., 2014). The increase of c-di-GMP due to *pleD* overexpression caused reduced swimming motility and an increase in tumor size, although histological sections of knots that developed in woody olive plants showed a reduction of the necrotic area (Perez-Mendoza et al., 2014). Intriguingly, no differences were observed in the production of IAA or the expression of the T3SS in this strain, suggesting an additional and yet unknown bacterial factor contributing to knot development that is controlled by PleD. Another study demonstrated the role of DgcP, a conserved DGC in pseudomonads, in knot development by Psv NCPPB 3335 (Aragón et al., 2015b). A mutant in *dgcP* caused approximately a three-fold decrease in the size of knots induced in olive plants (**Figure 2**) (Aragón et al., 2015b). Furthermore, the lack of *dgcP* led to an altered motile/sessile phenotype compared with the wild-type strain; it exhibited increased swimming motility and decreased biofilm formation, likely through changes in exopolysaccharide production (Aragón et al., 2015b). In the same study, a decrease in the expression of the T6SS genes *hcp1* and *vgrG* was observed in the *dgcP* mutant, although no significant differences were detected in the expression of the T3SS genes *hrpA*, *hrpL*, and *hopBL1* (Aragón et al., 2015b). On the other hand, the role of the previously described PDE BifA was evaluated with respect to tumor development in *P. savastanoi* (Aragón et al., 2015a). Deletion of *bifA* impaired swimming motility and decreased tumor size, although exopolysaccharide production and biofilm formation were not affected, which differs from results in other pseudomonads (Kuchma et al., 2007; Jimenez-Fernandez et al., 2015). In contrast, *bifA* overexpression reduced biofilm formation and exopolysaccharide synthesis, promoting swimming motility and positively regulating host invasion (Aragón et al., 2015a). Further studies on the molecular mechanisms underlying changes in cellular levels of c-di-GMP are necessary to fully comprehend how and when during the infection process this secondary messenger affects *P. savastanoi* virulence and tumor development.

## A WHOP Makes the Difference: Metabolism of Phenolic Compounds is Essential for *P. savastanoi* Induction of Full-Size Knots in Woody Hosts

Host adaptation and specialization of bacterial pathogens frequently involve gain, loss and evolution of virulence genes. Comparative genomic analyses among different bacterial strains constitute a fast and worthwhile approach for the identification of gene clusters putatively related to host adaptation. In the early 2010s, a 15 kb gene cluster was identified in Psv NCPPB 3335 (Rodríguez-Palenzuela et al., 2010) and in four strains of *P. syringae* pv. aesculi isolated from horse chestnut (Green et al., 2010), and found to be absent in the genomes of *P. syringae* strains infecting herbaceous hosts. Subsequent genomic analyses determined that the presence of this cluster, currently referred to as the WHOP region (from woody host and *Pseudomonas*) (Caballo-Ponce et al., 2017), is a feature exclusive to strains of phylogroups (PGs) 1 and 3 of the *P. syringae* complex isolated from woody organs of woody hosts, suggesting a specific role of this region in bacterial adaptation to woody tissues (Ramos et al., 2012; Bartoli et al., 2015; Nowell et al., 2016; Caballo-Ponce et al., 2017). In agreement with this hypothesis, the ability of several strains of the *P. syringae* complex, including Psv strains NCPPB 3335 and PseNe107, to grow endophytically in kiwi trees has been associated with codification of the *cat* operon (Bartoli et al., 2015).

A recent study delved into the genetic organization, function and role *in planta* of the WHOP region in Psv NCPPB 3335. Genes in this region are organized into four different operons (*catBCA*, *antABC*, *ipoABC*, and *dhoAB*) and three independently transcribed genes (*antR*, *benR* and a gene encoding a putative aerotaxis receptor). Although no function has yet been attributed to the *dhoAB* operon, the involvement of the Psv NCPPB 3335 *antABC* and *catBCA* operons in the catabolism of anthranilic acid and catechol, respectively, has been confirmed; additionally, the *ipoABC* operon was associated to an oxygenase activity acting on aromatic compounds (Caballo-Ponce et al., 2017). Nonetheless, the most intriguing conclusions of this work are derived from the role of the WHOP region during bacterial interactions with olive plants. While deletion of several WHOP genes had no effect on the size of knots induced by Psv NCPPB 3335 in micropropagated (i.e., non-woody) olive plants, mutations in the *antABC*, *catBCA*, or *ipoABC* operons in Psv resulted in virulence attenuation in woody olive plants (**Figure 2**). Additionally, a defect in competitive growth *in planta* was observed for the Psv *catBCA* and *dhoAB* mutants, as well as for the mutant affected in the putative aerotaxis receptor. However, this growth defect was exclusively observed in woody olive plants. Lignin, a complex organic network composed of different aromatic monomers (Boerjan et al., 2003; Ralph et al., 2004), accounts for up to 35% of the composition of wood (Pettersen, 1984). In fact, many lignin-related aromatic compounds are funneled into catechol and protocatechuate prior to their assimilation through the β-ketoadipate pathway (Harwood and Parales, 1996). While the machinery for protocatechuate degradation is widespread among *P. syringae* pathovars, the degradation of catechol into Krebs cycle intermediaries is generally restricted to strains of the *P. syringae* complex carrying the WHOP region (Caballo-Ponce et al., 2017). Hence, activities encoded in this region might provide additional pathways for the degradation of specific lignin-related compounds, allowing for the colonization of woody tissues by *P. savastanoi* and other strains of the *P. syringae* complex.

## Bacterial Communication Inside the Knots and the Influence of the Knot Microbiome on Knot Development

Although the study of bacterial plant diseases has traditionally focused on single pathogenic strains, in nature, most bacteria live as members of multispecies communities. To address this, the olive knot microbiome has recently been established as a model to study the role of interspecies bacterial communities in plant disease (Buonaurio et al., 2015). Metagenomic analysis of olive knots collected in Italy recently revealed the existence of a complex and diverse microbiome in this plant disease (Passos da Silva et al., 2014). The main constituents of this bacterial community are *Pseudomonas* spp. and *Pantoea* spp., with Psv making up almost 50% of the bacterial load. Although the proportion of other genera varied among samples, a common core composed of *Clavibacter*, *Curtobacterium*, *Enterobacter*, *Erwinia*, *Hymenobacter*, *Kineococcus*, *Pectobacterium*, and *Sphingomonas* was conserved in knots from different olive cultivars cultivated in diverse locations (Passos da Silva et al., 2014). Traditional culture methods have allowed identification at the species level of three non-pathogenic bacteria that co-reside with Psv within the olive knot, i.e., *Pantoea agglomerans* (Marchi et al., 2006), *Erwinia toletana* (Rojas et al., 2004) and *Erwinia oleae* (Moretti et al., 2011). Considering such biodiversity, it was reasonable to hypothesize that interspecies communication may occur within the olive knot and might influence the pathogenic behavior of Psv. In fact, three syntrophic pathways for the catabolism of plant-derived compounds have been predicted for the *E. toletana*-Psv consortium. For instance, the plant hormone salicylic acid, synthesized in response to a pathogenic infection (Loake and Grant, 2007), might be degraded via catechol by the cooperative action of these bacteria. In agreement with this hypothesis is the intimate co-localization of *E. toletana* and Psv within the olive knot, which may allow the interchange of public goods (Passos da Silva et al., 2014). Thus, combined metabolic features of Psv and *E. toletana* could both hamper plant defense responses against pathogens (easing the infection process) and diminish the toxicity of compounds generated during the process, leading to the increased bacterial populations observed in co-inoculation experiments (Hosni et al., 2011).

In addition to taking advantage of cooperative metabolism, interspecies cross-communication among bacteria occurs within the olive knot disease by sharing quorum sensing signals (Hosni et al., 2011). The quorum sensing system is a cell density-dependent gene regulatory network that relies on the production and detection of signal molecules, named acyl-homoserine lactones (AHLs) in Gram-negative bacteria, and that is often required for pathogenesis in plant diseases (Von Bodman et al., 2003). Interestingly, *E. toletana* DAPP-PG 735 and Psv DAPP-PG 722 produce the same types of AHLs (3-oxo-C6- and 3-oxo-C8-homoserine lactones). Production of AHL in Psv DAPP-PG 722 is a key feature in the normal generation of knots, as evidenced by the observation that a *luxI* mutant (i.e., unable to produce AHLs) of this strain induced only stem swelling in olive plants. Interestingly, co-inoculation of this Psv *luxI* mutant with *E. toletana* yielded a knot similar in size to that induced by the parental Psv strain, while restoration of knot size did not occur when the Psv *luxI* mutant was inoculated in combination with an *E. toletana luxI* mutant (Hosni et al., 2011). Similar pairwise inoculations were carried out between the Psv *luxI* mutant and a wild type *P. agglomerans* strain, resulting in only partial restoration of knot size. In fact, *P. agglomerans* DAPP-PG 734 synthesizes two types of AHLs (C4- and C6-homoserine lactones) that are structurally distinct from those produced by Psv (Hosni et al., 2011). Nevertheless, taking into account the versatility of the AHL-receptor proteins of the LuxR family, which are often capable of responding to structurally different AHLs (Subramoni and Venturi, 2009; Coutinho et al., 2013), the possibility that Psv reacts to the AHLs produced by *P. agglomerans* cannot be excluded. The quorum sensing regulon of this bacterial consortium, however, remains unknown.

## Additional Factors Involved in *P. savastanoi*-Induced Olive Knots

In addition to the aforementioned pathogenicity and virulence factors of *P. savastanoi* involved in knot formation, additional molecular mechanisms and metabolic pathways required for the full fitness and virulence of Psv in olive plants have been identified. Application of signature-tagged mutagenesis to Psv NCPPB 3335 during colonization of olive knots (Matas et al., 2012) identified the following: 18 genes involved in the biosynthetic pathways of nine of the 20 amino acids found in proteins, five genes related to the biosynthesis of the vitamins biotin, cobalamin, and thiamine, and three genes encoding putative citrate, sulfate and amino acid transporters. In addition to T3SS genes, this strategy also identified type II and IV secretion system genes as essential for full knot formation. Other Psv genes involved in knot formation and identified in this study, were deduced from mutants in a battery of genes involved in the tolerance and detoxification of reactive oxygen species, a set of genes required for the biosynthesis of the cell wall, a gene encoding a methyl-accepting chemotaxis protein, genes for several DNA-related proteins and numerous genes coding for proteins with unknown function (Matas et al., 2012). Although the roles of most of these additional factors in *P. savastanoi*-induced olive knots have not yet been studied in detail, this strategy provided confirmation of functional capabilities long believed to play a role in the virulence of *P. syringae* and related pathogens that have not yet been sufficiently examined.

## Concluding Remarks

Since the discovery of the bacterial etiology of certain plant galls, research on the molecular determinants of bacterial gall formation has predominantly focused on the soil and rhizosphere bacterial species *A. tumefaciens*, mainly due to its biotechnological relevance in plant breeding and transformation. Although diverse gall-forming bacteria shared essential determinants involved in gall formation, such as the production of phytohormones, various bacteria also use a distinctive set of molecular mechanisms to survive within the plant tissues and produce galls, which are dependent on their lifestyles and the specific plant tissues they infect. In this sense, *P. savastanoi* strains isolated from woody hosts exhibit a unique lifecycle among all other gall-forming bacteria, involving an epiphytic phase and an endopathogenic stage; the latter takes place predominantly inside trunks, stems and branches.

The discovery of the first mechanism involved in the generation of *P. savastanoi*-induced knots, the tryptophan-dependent IAA biosynthetic pathway via indole-3-acetamide, as well as the biosynthesis of IAA-Lys by certain *P. savastanoi* strains, dates back approximately four decades. However, the alternative IAA pathway remaining in Psv mutant strains lacking the *iaaMH* operon, as well as the function and role of the *iaaL* gene in this *P. savastanoi* pathovar, remains unresolved. Additionally, the role of the *ptz* gene in the biosynthesis of CKs is currently presumed only from experiments that involved the curing of *ptz*-encoding plasmids. Metabolomics analyses of the diversity of CKs and IAA-related compounds produced by *P. savastanoi ptz* and *iaaMH* mutants, respectively, are required to identify alternative biosynthetic pathways for these phytohormones.

Bioinformatics analyses of the putative T3E repertoires encoded in the genomes of diverse *P. savastanoi* strains have recently defined a core of approximately 20 T3Es shared among *P. savastanoi* pathovars isolated from diverse hosts. Further comparative analyses using a higher number of genomes are necessary to determine the specific T3Es encoded by strains isolated from diverse hosts and to approach their role during the infection of woody hosts.

The recent discovery of the role of c-di-GMP in the formation of Psv-induced knots in olive plants leads to further exploration and functional analysis of the large number of DGC and PDE proteins encoded in the genomes of *P. savastanoi* strains, with the aim of establishing their role in the lifestyle of this pathogen. The identification of the specific targets of these enzymes would also help us ascertain the signaling pathways interconnecting c-di-GMP with other bacterial processes contributing to virulence, and also to establish their role in knot formation. Since the discovery of the WHOP region in the genome of Psv and *P. syringae* pv. aesculi in the early 2010’s, a role of this genomic cluster in the adaptation of bacteria from the *P. syringae* complex to woody hosts has been suggested by several authors. Despite emerging evidences supporting this hypothesis over the last 2 years, the specific function of the WHOP region during the colonization of woody organs remains to be elucidated. Several functions have been suggested for the WHOP-encoded activities that might explain the advantage conferred by this region to *P. savastanoi*, including, (i) the assimilation of lignin-related compounds, (ii) the modification of certain compounds to decrease their toxicity, and (iii) interference with the IAA pool, influencing virulence and thus knot size. However, all these hypotheses remain to be critically tested. Further studies are also needed to fully understand the mechanisms involved in the interspecies synergism established within the microbial community in knots. To this end, metagenomic analyses of knot microbiomes from diverse hosts might help to generate new hypotheses that aid in our understanding of the signaling pathways shared among diverse microbial species and the possible metabolic complementarity established among them.

A great deal of research is still necessary to establish approaches to understand the evolution and adaptation of *P. savastanoi* to novel hosts and to develop effective control strategies for *P. savastanoi*-induced knot diseases. We perceive the following priority areas for future research on the interaction of *P. savastanoi* with woody hosts:

(1) The dissection of all metabolic routes for the biosynthesis of phytohormones and of the resulting biologically active compounds. This will facilitate the identification of the likely role of phytohormones in the regulation of virulence genes, the suppression of plant defense responses and the survival of *P. savastanoi*, both during infection and epiphytically.
(2) The identification of T3SS effectors specifically required for the infection of woody hosts, and the determination of their role in host specificity.
(3) The discovery of new types of genes that deeply influence virulence is changing our understanding of pathogenicity and the mechanisms underlying host specificity. Research in the many roles of c-di-GMP in virulence, and the specific functions of the WHOP region should illuminate the infection process and the contribution of plant phenolics to defense.
(4) The simplification of pathosystems has been critical for the dissection of pathogenicity, but is increasingly clear that the interactions between plants and pathogens are influenced by a potentially diverse community of microorganisms. The metagenomics analyses of these communities will propel our understanding of a further organizational level that, ultimately, determines whether the outcome is disease or a plentiful harvest.

## Author Contributions

All authors contributed to manuscript writing and elaboration of Figures and Tables.

## Conflict of Interest Statement

The authors declare that the research was conducted in the absence of any commercial or financial relationships that could be construed as a potential conflict of interest.
